# Malignant peripheral nerve sheath tumor associated with neurofibromatosis type 1, with metastasis to the heart: a case report

**DOI:** 10.1186/1746-1596-5-2

**Published:** 2010-01-09

**Authors:** Masanori Kitamura, Naoki Wada, Shigenori Nagata, Norishige Iizuka, Yu-Fen Jin, Miki Tomoeda, Michiko Yuki, Norifumi Naka, Nobuhito Araki, Chikao Yutani, Yasuhiko Tomita

**Affiliations:** 1 Department of Pathology, Osaka Medical Center for Cancer and Cardiovascular Diseases, 1-3-3 Nakamichi, Higashinari, Osaka, 537-8511, Japan; 2 Department of Pathology, Osaka University Graduate School of Medicine, 2-2 Yamadaoka, Suita, Osaka, 565-0871, Japan; 3 Department of Orthopedic surgery, Osaka Medical Center for Cancer and Cardiovascular Diseases, 1-3-3 Nakamichi, Higashinari, Osaka, 537-8511, Japan; 4 Department of Life Science, Okayama University of Science, 1-1 Ridaicho, Kita, Okayama, Okayama, 700-0005, Japan

## Abstract

A rare case is presented of a 61-year-old man with a malignant peripheral nerve sheath tumor associated with neurofibromatosis type 1, with metastasis to the heart. The primary tumor originated in the right thigh in 1982. Since then, the patient has had repeated local recurrences in spite of repeated surgical treatment and adjuvant chemotherapy. He has developed previous metastases of the lung and heart. The patient died of cardiac involvement.

## Background

Malignant peripheral nerve sheath tumor (MPNST) is an aggressive and uncommon neoplasm that develops within a peripheral nerve; most cases of which are associated with neurofibromatosis type 1 (NF1). Metastasis of MPNST usually occurs in the lung [1], whereas cardiac metastasis of MPNST is quite rare [2-4]. In this report, we describe a case of MPNST that metastasized to the heart, with a review of the literature.

## Case Presentation

**Figure 1:**
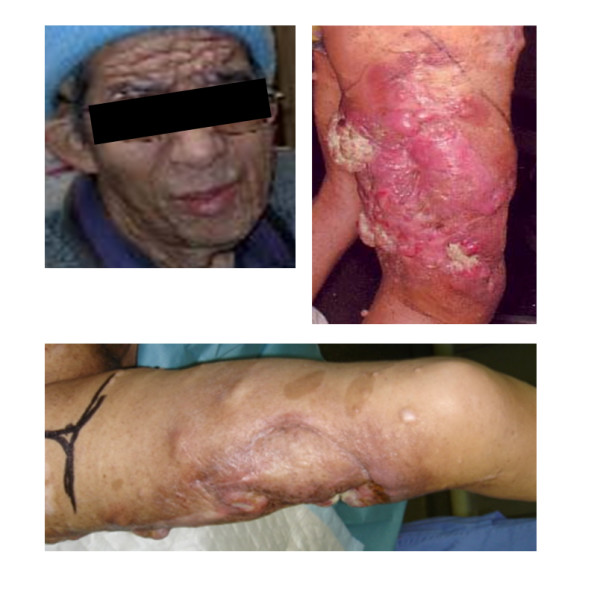
**Clinical manifestations of NF1: multiple cutaneous neurofibromas and café-au-lait spots**.

**Figure 2:**
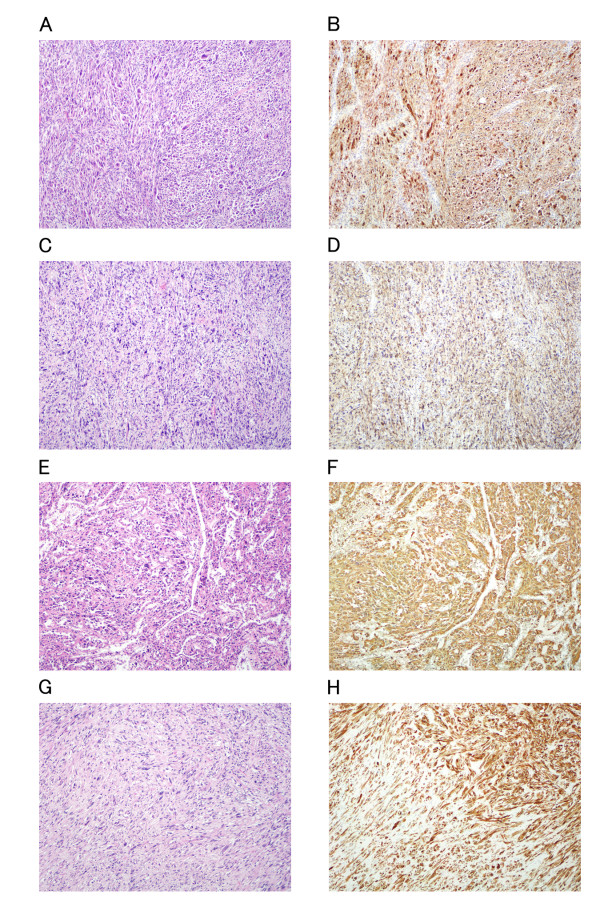
**Hematoxylin and eosin and S-100 staining of the tumors of the thigh treated surgically in 1990 (A, B), recurrent tumor operated in 1995 (C, D) and metastatic tumor in heart (E, F) and lung (G, H) at autopsy in 2007**. A, C, E, G: Hematoxylin and eosin staining; B, D, F, H: S-100 immunohistochemistry. Histological factors such as cellularity, degree of nuclear atypia, mitotic counts, and S-100 immunoreactivity were similar among the specimens.

**Figure 3:**
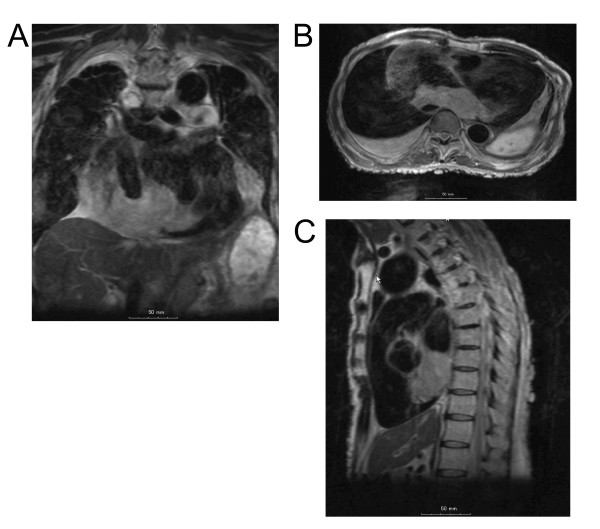
**Magnetic resonance imaging showed a large tumor in the inferior-posterior wall of the heart**. (A) Frontal cross section. Size: 7.5 cm. (B) Horizontal cross section. Size: 9 cm. (C) Sagittal cross section. Size: 6.5 cm.

**Figure 4:**
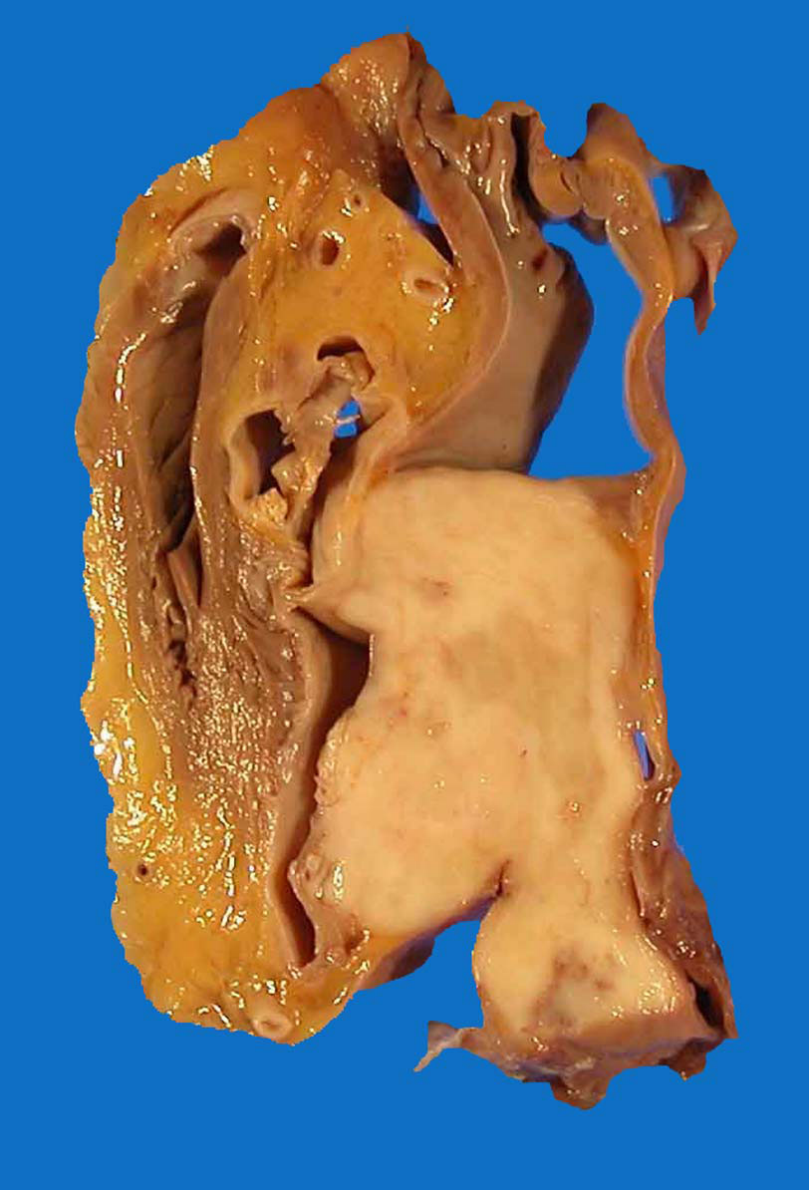
**Autopsy specimen showing a tumor in the heart**.

A 61-year-old man presented with clinical stigmata of NF1. Genetic analysis had not been performed. He had no family history and had developed multiple neurofibromas and café-au-lait spots on his body (Figure 1). He underwent surgical treatment for a growing tumor (details are unknown) on the posterior surface of his right thigh at another hospital in April 1982, and was referred to our institution because of regrowth of the tumor in October 1990. Wide resection was performed in November 1990 (Figure 2). The histological diagnosis was MPNST developed on a background of NF1, and he received adjuvant chemotherapy. Since 1990, the patient has had repeated local recurrences (7 times, until May 2003) and has received surgical treatment for each recurrent tumor. In December 2003, the patient presented with a skin tumor of the head, and the lesion was resected. The histological diagnosis was MPNST that arose in a neurofibroma of the head skin. Two years later, the right leg was amputated at the hip joint because of local recurrence of the leg lesion, with aggressive growth and rupture. Computed tomography (CT) performed in August 2005 showed multiple lung lesions, suggestive of tumor metastases. Some of the lung lesions were resected surgically in January 2006 and diagnosed histologically as MPNST metastases. After the operation, he developed new lung metastases. In May 2007, he was admitted to our institution with sudden dyspnea and palpitations. Echocardiography revealed an enlarged heart and cardiac effusion, which did not suggest cardiac tamponade. Magnetic resonance imaging showed the presence of a large tumor (9 cm) in the inferior-posterior wall of the heart (Figure 3). CT revealed an infarction of the inferior lobe of the left lung, which was derived from tumor emboli. As a result of the worsening condition, irradiation was applied to the cardiac mass (2 × 25 Gy). The patient's condition still worsened, with increasing cardiac effusion and arrhythmia. Regardless of drainage of the effusion, he died of acute heart failure soon after. At autopsy, the cardiac mass was confirmed histologically as MPNST (Figure 4). The cardiac lesion was thought to be a metastatic tumor, because neurofibroma was not seen as possible precursor within the lesion. Serial sections suggested that the tumor involved the conduction system. MPNST affected both lungs. Histological images of cardiac and pulmonary tumor were similar to those of the tumor of the right leg that was amputated in 2005 (Figure 2).

## Discussion

Patients with NF are at greatest risk for developing sarcomas, including MPNST. The incidence of MPNST arising in NF is 4.6%, which is much higher than the 0.001% in general population [5]. The most common metastatic site of MPNST is the lung [1,4]. Cardiac involvement from metastatic MPNST is extremely rare, whether with or without a background of NF [2-4]. Our patient is one of the extremely rare cases of cardiac metastasis of MPNST associated with NF1. Most of the cardiac metastases are preceded by other metastatic lesions, such as in the lung. Although the possibility of cardiac involvement becomes higher as the tumor progresses, details of the histopathological features specific to cardiac metastasis remain to be investigated. Recent studies have in part revealed the genomic imbalance in sporadic and NF1-associated MPNST [6,7]. The biology of metastatic features of MPNST, however, is still unknown.

The prognosis of patients with MPNST is generally poor. Aggressive surgery significantly improved disease-free survival [5,8]. Adjuvant chemotherapy and radiotherapy has not been proven to prolong patient survival, but it is effective as a palliative option [4,8,9]. If clinical symptoms of cardiac dysfunction occur during the progression of MPNST, it might be that the heart is involved. In such cases of MPNST, it is necessary to exclude cardiac involvement, even if it is rare, by occasional echocardiography. Early diagnosis can allow timely surgical intervention, if the patient is operable, which may improve results, as in the case described here.

Clinical features vary according to the site of cardiac involvement[10], such as pericardium, epicardium, myocardium or endocardium. The present case showed a large mass in the myocardial region, which was accompanied with increasing pericardial effusion and arrhythmia. Serial histological sections revealed that the metastatic tumor markedly affected the common bundle of His, in addition to the ordinary myocardium. Based on these findings, we surmised that complete atrioventricular block was attributable to cardiac metastasis of MPNST, which is causative of circulatory failure [10]. Related features of cardiac involvement of MPNST are uncertain. More cases should be reported to elucidate the clinical entity associated with cardiac involvement of MPNST and to formulate an appropriate treatment strategy.

Most NF1 patients carry a constitutional mutation of the *NF1 *tumor suppressor gene [11]. Biallelic inactivation of *NF1 *and mutations of numerous additional tumor suppressor genes within the *p19*^*ARF*^-*MDM2-TP53 *and *p16*^*INK*4*A*^-*Rb *signaling cascades have been identified in MPNSTs [12,13]. These abnormalities of suppressor genes, except for *NF1*, are not present in neurofibromas. It is therefore thought that the development of neurofibromas and their subsequent progression to become MPNSTs involves a sequential series of tumor suppressor mutations. Deletions and other mutations that result in loss of function of the *TP53 *tumor suppressor gene are some of the more common abnormalities found in MPNSTs. Biallelic inactivation of the *TP53 *locus is found rarely in MPNST, which has led to the suggestion that hemizygous *TP53 *mutations may suffice for neurofibromas to progress and become MPNSTs.

A recent study has demonstrated that two MPNST cell lines derived from sporadically occurring MPNSTs have functional and intact *NF1 *genes [14]. Paradoxically, however, microarray studies that have compared the transcriptomes of sporadic and NF1-associated MPNSTs have not found a molecular signature that distinguishes these neoplasms [14,15].

For understanding of these complex neoplasms and the development of the effective new therapy, further investigation will be needed into the clinical features and the basic science.

## Conclusion

Although cardiac involvement of MPNST is rare, precise examination including occasional echocardiography is necessary when clinical signs of tumor development in the heart are suspected.

## Consent

Written informed consent was obtained from the patient's family for publication of this case report and any accompanying images. A copy of the written consent is available for review by the Editor-in-Chief of this journal.

## Competing interests

The authors declare that they have no competing interests.

## Authors' contributions

MK participated in the pathological examination of the case, the design of the study and drafting the manuscript.

NW, SN, NI and CY participated in the pathological examination of the case.

YFJ, MT, and MY participated in the immunohistochemical analysis.

NN and NA participated in collecting clinical data and images.

YT participated in its design and coordination and helped to draft the manuscript.

All authors have read and approved the final manuscript.
